# Environmental stress, water inequality, and environmental justice in Palestinian urban areas: a GIS-based assessment of sanitation and infrastructure disparities in the southern West Bank

**DOI:** 10.1186/s12940-026-01286-8

**Published:** 2026-03-16

**Authors:** Nidal Nassar, Hamzeh Al Zabadi, Zohra Chabaane

**Affiliations:** 1 National Agronomic Institute of Tunisia, Laboratory GREEN-TEAM LR17AGR01, University of Carthage, 43 Avenue Charles Nicolle, Mahrajène, Tunis, 1082 Tunisia; 2 Institution of Agricultural Research and Higher Education (IRESA), National Agronomic Institute of Tunisia, Laboratory GREEN-TEAM LR17AGR01, 43 Avenue Charles Nicolle, Mahrajène, Tunis, 1082 Tunisia; 3 Department of Public Health, Faculty of Medicine and Health Sciences, An-Najah National University, An-Najah National University Palestine, Nablus, Palestine

**Keywords:** Environmental Justice, Environmental Stress Index (ESI), GIS, Dura, Al-Fawwar Refugee Camp, Nahal Negohot Settlement, Hebron, West Bank, Palestine

## Abstract

This study examines environmental justice disparities in the southern West Bank by assessing groundwater quality, wastewater management, and water supply infrastructure in Dura City, Fawwar Refugee Camp, and the Israeli settlement of Nahal Negohot. A mixed-methods approach combined field-based water sampling (71 points) with socio-demographic data (2014–2024) and GIS-based spatial analyses to generate composite indices, including the Environmental Stress Index (ESI) and Pollution Risk Index (PRI).

Results indicate pronounced spatial inequities. Nitrate (NO₃⁻) concentrations in Dura City ranged from near zero to extreme peaks of 1,667.5 mg/L, exceeding WHO standards by up to 34 times, while Fawwar Camp exhibited moderate exceedances (51.5–147.1 mg/L). Nahal Negohot maintained low nitrate levels (<50 mg/L) due to centralized treatment and secure water supply. Total dissolved solids (TDS) reached 2,100 mg/L in Dura, 724 mg/L in Fawwar, and remained below 250 mg/L in Nahal Negohot. Electrical conductivity (EC) followed similar patterns, with values exceeding 2,600 µS/cm in Dura, 1,500 µS/cm in Fawwar, and <500 µS/cm in Nahal Negohot. The PRI highlighted high pollution risk in Dura (4.05), moderate risk in Fawwar (2.0–3.5), and low risk in Nahal Negohot (1.35–2.0).

Water allocation in 2024 revealed stark inequities: Dura and Fawwar received only 13.5% and 7.2% of total supply, respectively, corresponding to 64.6 and 53 L/person/day, while Nahal Negohot received 79.3%, equivalent to 650 L/person/day. The ESI indicated high environmental stress in Dura, moderate stress in Fawwar, and low stress in Nahal Negohot, reflecting cumulative pressures from poor infrastructure, unregulated wastewater, illegal water extraction, network losses, and administrative deficiencies in Palestinian areas.

These findings indicate that groundwater contamination and unequal water distribution are intertwined with structural and administrative constraints, resulting in environmental injustice. The study emphasizes the urgent need to enhance the capacity for integrated water and wastewater management, strengthen monitoring, and implement governance reforms to promote equitable and sustainable access to resources in the study area.

## Introduction

Palestinian urban areas are increasingly exposed to escalating environmental pressures resulting from rapid population growth, unregulated urban expansion, and often limited capabilities and poor management. The Oslo II Agreement [13] divided the West Bank into Areas A, B, and C, directly impacting environmental governance. Areas A and B are under Palestinian administration, where infrastructure development does not require prior Israeli approval. In contrast, Area C is under Israeli administrative control, requiring prior approval for any infrastructure development, particularly concerning drinking water or wastewater treatment (Oslo II [13]).

These interacting factors have generated structural patterns of environmental inequality, manifesting in disparities in water quality, sanitation services, and air conditions between Palestinian communities and adjacent Israeli settlements. Such environmental deterioration underscores the urgent need for integrated scientific frameworks capable of spatially monitoring environmental changes, identifying vulnerable hotspots, and supporting equitable and sustainable resource management. This need is particularly critical for general environmental conditions, the provision of safe drinking water, and the management of wastewater systems, in line with United Nations recommendations for safeguarding public health [21].

The Hebron region, specifically Dura City, Al-Fawwar Refugee Camp, and the Nahal Negohot settlement, provides a microcosm of structural environmental injustice in the West Bank. Despite their geographic proximity, these areas exhibit pronounced disparities in infrastructure, service quality, and institutional capacity. Palestinian communities largely rely on decentralized and often inadequate water and sanitation systems, whereas the neighboring Israeli settlement benefits from highly efficient centralized infrastructure. These asymmetries form a critical basis for examining the relationship between institutional inequality and environmental degradation.

Access to water and its quality are central to these disparities, as Palestinian communities face restrictions on developing water infrastructure, which has driven Palestinian residents to seek alternatives such as springs and water wells that are vulnerable to pollution from agricultural runoff and sewage leakage from widespread seepage pits among the population. Meanwhile, Israeli settlements receive preferential allocation under occupation-related policies [15, 16]. Prevailing climatic conditions—characterized by high temperatures, elevated evaporation rates, annual rainfall ranging between 300–700 mm, and humidity levels of 70–80% in mountainous and semi-coastal areas—further intensify competition over limited water resources [14].

This study develops a comprehensive methodological framework to assess environmental stress in Palestinian urban contexts through the integration of Geographic Information Systems (GIS), spatial analysis, and multi-criteria decision-making models. By combining physical, social, and institutional variables, the framework enables the identification of environmental hotspots, the evaluation of cumulative stress on local communities, and the prediction of future degradation trends under persistent administrative and infrastructural constraints [11, 20]. The findings aim to contribute to equitable environmental governance, sustainable urban planning, and fair resource management in contexts marked by systemic inequalities.

## Literature review

### Water quality

Groundwater quality assessments are critical for understanding environmental and public health risks. Sirajudeen et al. [12] evaluated groundwater in the Ampikapuram area (India) using the Water Quality Index (WQI), highlighting elevated levels of nitrate, TDS, chloride, and other physicochemical parameters, which indicate poor water quality requiring treatment before consumption. Although geographically distinct, these findings illustrate general principles of groundwater vulnerability to chemical contamination. In the West Bank context, elevated nitrate and TDS levels in Palestinian localities, as documented in multiple field studies, suggest similar environmental pressures from untreated wastewater, agricultural runoff, and over-extraction [5, 8, 22].

### Water Infrastructure

Infrastructure capacity strongly influences water quality and accessibility. Bill Kingdam et al. [9] emphasized the role of private sector participation, performance-based contracts, effective tariffs, and regulatory standards in minimizing water loss. Lambert and Taylor [7] further highlighted the importance of monitoring non-revenue water as an indicator of network conditions, stressing that systematic reporting is essential for effective management. In the Palestinian context, Murar et al. [1, 2] demonstrated that institutional structure affects service efficiency, with utilities outperforming municipalities, and that regional grouping of providers can reduce costs. Alkelani and Awad [5] recommended network rehabilitation, alternative water sources, pump capacity enhancement, and prepaid meters to curb illegal water use. Saleh and Rattroot [17] showed that predictive modeling, including LRSVM, can anticipate pipe failures and mitigate losses, while Al Edie and Qtaishat [4] highlighted the role of GIS for network monitoring and reducing unauthorized consumption.

Infrastructure vulnerabilities in Palestinian areas are exacerbated by physical damage to networks, unauthorized water extraction, and losses due to leakage or poor maintenance, which are compounded by limited municipal capacity and restrictions under the Oslo II Agreement [6, 22]. Equipment and electricity shortages further hinder performance during emergencies [3], and the absence of centralized monitoring reduces the effectiveness of mitigation strategies.

### Environmental justice

Environmental justice frameworks provide insight into disparities in water access and risk exposure. Selby [18] argues that institutional arrangements such as the Joint Water Committee maintain Israeli control over water resources, producing distributive, procedural, and recognition injustices. Weinthal & Sowers [23] demonstrate how restrictions on Palestinian infrastructure generate environmental inequalities, increasing risks from contaminated water and inadequate sanitation. Trottier [19] and Zeitoun [25] further highlight that Israeli dominance over water distribution and governance structures limits Palestinian participation in decision-making, shaping uneven exposure to environmental hazards. These studies suggest that water quality and infrastructure challenges in the West Bank are not merely technical problems but are deeply embedded in political and structural inequities.

### Conflict context

Political and military constraints in the West Bank significantly shape water access and quality. Restrictions on infrastructure development in Areas B and C, combined with permit limitations and administrative control, reduce the capacity of Palestinian municipalities to maintain or expand water networks. Unauthorized water extraction by residents and damage to existing networks, often occurring in response to shortages or inequitable distribution, further compound environmental pressures. These dynamics create a feedback loop where infrastructure deficiencies and governance constraints jointly exacerbate environmental stress and public health vulnerabilities [18, 23].

### West Bank focus

Geographically, studies converge on the southern West Bank, particularly Dura City and Fawwar Refugee Camp, as sites facing acute water quality and infrastructure challenges. Water is primarily sourced from Mekorot and distributed through the West Bank Water Department. However, disparities in allocation, network maintenance, loss prevention, and distribution management indicate that population size and governance structures significantly influence access. In contrast, the Israeli settlement of Nahal Negohot in the same region demonstrates relatively low environmental stress and high-quality water provision, highlighting the spatial dimension of inequality and the intersection of technical and administrative factors [4, 25].

## Methodology

### Study area description

#### Geographical Scope

The study area includes three distinct localities located in the southern part of the West Bank within Hebron Governorate, representing a spectrum of socio-political, infrastructural, and environmental conditions. Dura City is a Palestinian municipality characterized by moderate urban expansion, where wastewater management primarily relies on decentralized septic tank systems. In contrast, Fawwar Refugee Camp is a densely populated area with limited public infrastructure and no centralized wastewater treatment facilities. Nahal Negohot, an Israeli settlement, is equipped with centralized wastewater infrastructure and benefits from a continuous, high-volume water supply.

Although these localities are geographically adjacent, they differ substantially in infrastructure availability, environmental management systems, and exposure to environmental stressors. The three sites were strategically selected to represent a microcosm of broader environmental justice dynamics in the Palestinian territories, particularly the systemic disparities between Palestinian communities and Israeli settlements in access to clean water, sanitation services, air quality conditions, and environmental monitoring and enforcement mechanisms.

Spatial boundaries were obtained from official shapefiles and analyzed using ArcGIS Pro to examine geospatial relationships between infrastructure distribution, pollution indicators, and demographic patterns.

#### Socio-demographic characteristics

The selected localities display notable differences in demographic structure and population density, which directly influence the environmental pressures observed in each area. Dura City had an estimated population of 47,780 in 2024 (Dura [10]), reflecting steady growth over the past decade that has increasingly strained water infrastructure and decentralized wastewater systems. Fawwar Refugee Camp recorded an estimated population of 9,056 in 2024, according to estimates and calculations by the camp administration and UNRWA in 2023, with the population concentrated within a limited built-up area. As one of the most densely populated areas in the governorate, the camp continues to face chronic infrastructural limitations and limited-service provision. Nahal Negohot had an estimated population of approximately 425 residents in 2024, based on aggregated geographic and administrative data sources (unpublished data, 2024). This settlement is characterized by low population density, planned housing units, and consistent access to public utilities and environmental services, resulting in a relatively lower per capita environmental impact. Population figures were compiled from time-series municipal and administrative datasets spanning 2014–2024, integrating official estimates from PCBS, UNRWA, and local municipal records. It should be noted that precise official statistics for Israeli settlements are not publicly available, and the figures for Nahal Negohot should be interpreted as approximate estimates.

#### Infrastructure profile

Infrastructure conditions across the study area vary considerably, particularly in relation to water supply systems, wastewater management, and environmental protection mechanisms. For analytical purposes, the locations can be broadly grouped into Palestinian localities (Dura and Fawwar) and the Israeli settlement of Nahal Negohot.

In Dura and Fawwar, water supply is intermittent and limited, with 2024 per capita availability estimated at 64.6 L/day and 53 L/day, respectively [15], values that remain below WHO recommended guidelines. Wastewater in both areas is managed through decentralized systems, primarily septic tanks and cesspits, without connection to centralized treatment facilities. Solid waste management services are often fragmented, with instances of open dumping and occasional burning observed.

By contrast, Nahal Negohot receives a continuous water supply of approximately 650 L/day per capita [15] and is supported by a centralized wastewater treatment system and formally structured solid waste management services.

This infrastructural disparity carries significant implications for environmental quality and public health. Reliance on septic systems may contribute to groundwater contamination, particularly where maintenance is inadequate or system design is suboptimal. Leakage from such systems can result in elevated concentrations of nitrates, nitrites, and chlorides in underlying aquifers, thereby posing risks to drinking water quality. Furthermore, the absence of centralized wastewater infrastructure in many Palestinian localities increases environmental vulnerability and limits the capacity of local authorities to implement effective mitigation and monitoring strategies.

#### Political and institutional context

The environmental and infrastructural disparities across the study locations are deeply influenced by the broader political structure governing the West Bank. Under the Oslo II Interim Agreement [13], the West Bank was divided into Areas A, B, and C, leading to a fragmented governance system. While Dura City and Fawwar Camp fall within Areas A and B, infrastructure development—particularly in critical sectors like water and wastewater—often requires Israeli approval when it extends into or depends on access through Area C, which remains under full Israeli control. This has created persistent barriers to planning, constructing, and maintaining environmental infrastructure in Palestinian communities.

In contrast, Israeli settlements such as Nahal Negohot operate within a unified legal and administrative framework under Israeli jurisdiction. These areas benefit from direct integration into national water, wastewater, and environmental management systems, bypassing the regulatory and logistical constraints faced by surrounding Palestinian localities. This political-institutional asymmetry contributes directly to the infrastructure and environmental disparities examined in this study.

### Research design

This study employs a comparative mixed-methods research design to examine environmental stress and justice disparities across three adjacent but socio-politically and infrastructurally distinct localities in southern West Bank: Dura City, Fawwar Refugee Camp, and Nahal Negohot settlement. The mixed-methods approach integrates quantitative measurements, qualitative contextual understanding, and GIS-based spatial analysis, providing a holistic framework to assess environmental inequalities.

#### Rationale for the mixed-methods design

A mixed-methods design was adopted because environmental justice constitutes a multi-dimensional issue that encompasses both measurable environmental disparities—such as water quality, infrastructure provision, and pollution levels—and structural, political, and governance factors that cannot be adequately captured through quantitative data alone.

The quantitative component focuses on measuring physical and environmental variables, including water quality parameters, air quality indicators, population density, and per capita water supply. In parallel, the qualitative and contextual dimension examines socio-political and institutional factors, such as governance fragmentation under the Oslo II framework, infrastructural constraints, and regulatory asymmetries.

GIS and spatial analysis serve as an integrative framework that combines spatial, temporal, and environmental datasets to visualize disparities, identify environmental hotspots, and support multi-criteria assessments.

By integrating these complementary approaches, the study ensures that findings are empirically grounded, contextually interpreted, and spatially explicit, thereby strengthening the robustness of the environmental justice assessment.

#### Quantitative data collection and analysis

Field-based water sampling was conducted at 71 locations, including 38 sites within Palestinian localities and one site in Nahal Negohot, in addition to supplementary reference points. The analysis measured key physicochemical parameters, including pH, electrical conductivity (EC), total dissolved solids (TDS), nitrates, ammonium, phosphates, chlorides, potassium, sodium, and sulfates.

Water supply and population data were compiled from time-series records covering the period 2014–2024 to calculate per capita water availability and assess temporal trends in distribution patterns. Statistical analyses included descriptive statistics, Pearson correlation analysis, and inferential tests such as ANOVA or Kruskal–Wallis, depending on normality assumptions. Measured values were compared against World Health Organization (WHO) guidelines to evaluate compliance and potential health risks.

#### GIS-Based spatial analysis

Administrative and infrastructure layers—including boundaries, built-up areas, road networks, and educational facilities—were integrated with environmental datasets using ArcGIS Pro. Spatial joins, raster-based analyses, and interpolation techniques were applied to construct composite spatial indicators. These included the Environmental Stress Index (ESI), which integrates air quality, water quality, and population density variables, and the Pollution Risk Index (PRI), which combines pollutant concentrations with hydrological flow patterns and terrain susceptibility factors.

Furthermore, hotspot analysis using the Getis-Ord Gi* statistic was performed to identify statistically significant clusters of elevated environmental risk, thereby supporting spatial prioritization for targeted interventions.

#### Integration of components

Quantitative data provide objective measurements of environmental conditions and enable statistical assessment of disparities in water quality, infrastructure provision, and environmental exposure. Complementing this, qualitative and contextual interpretation helps explain systemic inequalities and structural political constraints that shape environmental outcomes. GIS analysis further strengthens the framework by visualizing spatial patterns and integrating multiple datasets to construct composite indices, including the Environmental Stress Index (ESI) and the Pollution Risk Index (PRI).

This methodological triangulation ensures that the findings are not only statistically robust but also contextually grounded and spatially explicit, thereby offering a comprehensive assessment of environmental justice dynamics within the study area.

### Data sources and collection

#### Field-based water quality sampling

**Fig. 1:**
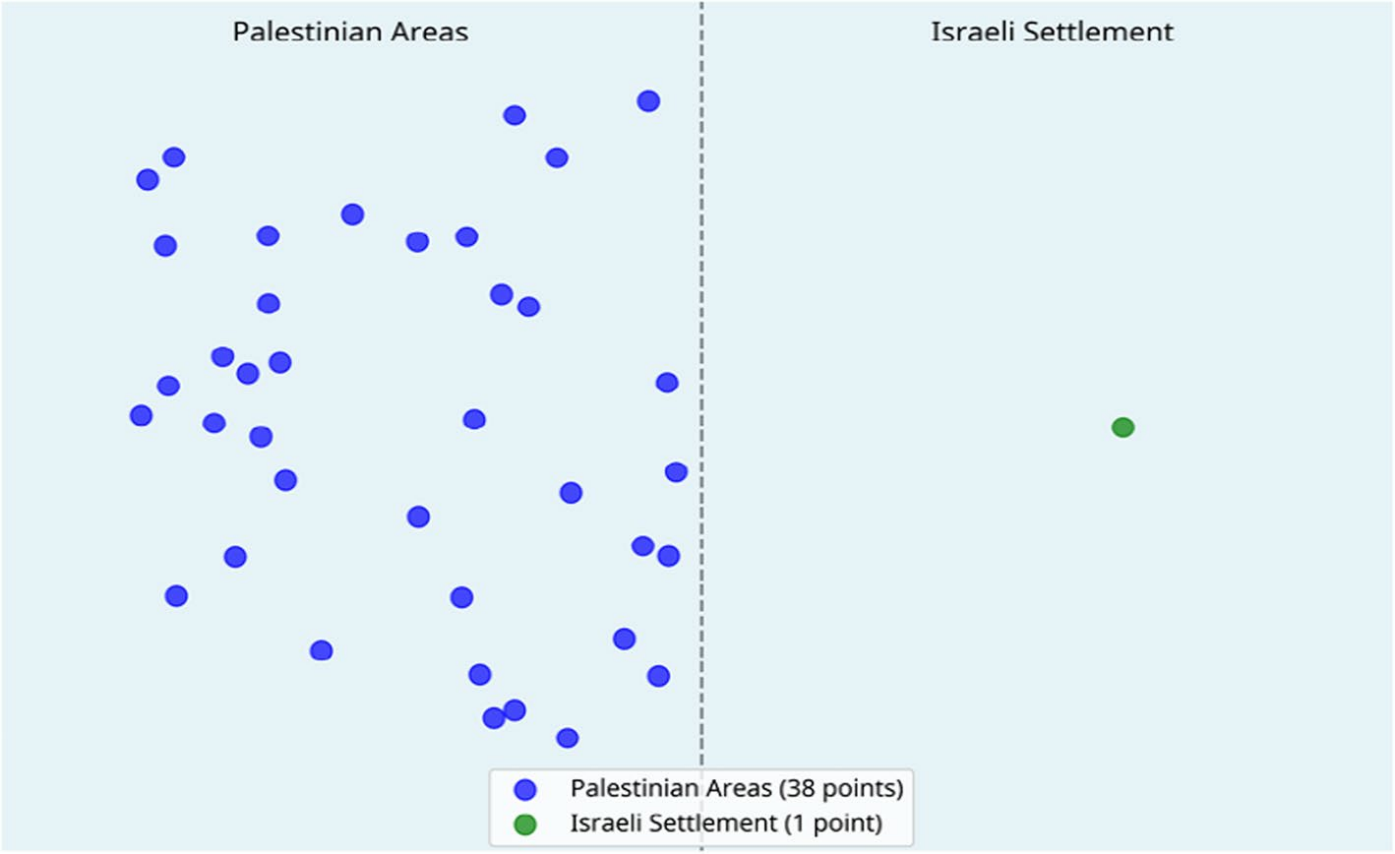
Water quality monitoring point

Water quality data were collected through field-based sampling across 71 monitoring points, strategically distributed to capture environmental variability across the study locations. The distribution included 38 points in Palestinian localities (Dura City and Fawwar Refugee Camp), 1 point in the Israeli settlement of Nahal Negohot, and additional reference points in surrounding areas. As illustrated in Fig. 1 below, the spatial distribution of sampling sites was designed to reflect varying land uses and potential pollution sources.

Each water sample was analyzed for a comprehensive set of physicochemical parameters, including pH, electrical conductivity (EC), total dissolved solids (TDS), nitrate (NO₃⁻), ammonium (NH₄⁺), phosphate (PO₄^3^⁻), chloride (Cl⁻), potassium (K⁺), sodium (Na⁺), and sulfate (SO₄^2^⁻). These parameters were selected due to their relevance in assessing groundwater quality, potential contamination sources, and public health risks.

Strict quality assurance and quality control (QA/QC) procedures were applied throughout the sampling process. Field instruments were calibrated prior to each sampling session using NIST-traceable standards to ensure measurement accuracy. Duplicate samples were collected at 10% of monitoring locations, and field blanks were employed to detect potential contamination during sample handling and transport. In addition, a detailed chain-of-custody protocol was maintained from sample collection through laboratory analysis to preserve sample integrity and analytical reliability.

#### Administrative and socio-infrastructure data

To support the spatial analysis and interpretation of environmental stress across the study locations, a comprehensive set of administrative and socio-infrastructure datasets was compiled. Shape files delineating the boundaries of Dura City, Fawwar Refugee Camp, and Nahal Negohot settlement were acquired to define the geographical scope. Supplementary spatial layers, including schools, built-up areas, road networks, and population density, were incorporated to facilitate spatial correlations between demographic, infrastructural, and environmental variables. Time-series data (2014–2024) on population and water supply were sourced from municipal records, organized by locality and year, and linked to spatial features using standardized location codes. Per capita water supply was calculated and evaluated against World Health Organization [24] thresholds to assess adequacy. These datasets were integrated into an ArcGIS Pro geodatabase, enabling multi-layer spatial analysis to support the construction of the Environmental Stress Index (ESI) and the identification of environmental hotspots.

#### Spatial data processing

Spatial and tabular datasets were integrated using ArcGIS Pro to enable combined spatial and statistical analysis. Spatial joins were performed to aggregate water quality parameters, air quality data, and population figures into the boundaries of each study locality. For variables with multiple sampling points per area, one-to-many joins were used to compute mean values per parameter.

All quantitative indicators were standardized using Min–Max normalization, based on the formula:

$${X}_{norm }= \frac{X-{X}_{min}}{{X}_{max}-{X}_{min}}$$

A total of 14 water quality parameters, along with Air Quality Index (AQI) values and population, were normalized. Parameters with a negative environmental impact (e.g., AQI, NO₃⁻, EC, TDS, Cl⁻) were normalized directly, while positive indicators (e.g., Dissolved Oxygen, vegetation cover) were inversely normalized to maintain interpretative consistency.

The processed and normalized layers formed the basis for the development of composite indices and spatial classification maps used in subsequent analyses.

### Analytical methods

#### Water quality statistical analysis

Water quality data from 71 sampling points were analyzed using descriptive, correlational, and inferential statistical methods. Descriptive statistics (mean, median, range) were calculated for all parameters to explore spatial trends and identify anomalies.

Pearson correlation analysis was applied to detect relationships between variables such as NO₃⁻, EC, and Cl⁻, which may indicate shared contamination sources. Group comparisons between Palestinian areas (Dura and Fawwar) and the Israeli settlement (Nahal Negohot) were conducted using ANOVA or Kruskal–Wallis tests, depending on data distribution characteristics.

Measured values were also compared against World Health Organization (WHO) drinking water quality guidelines, which provide internationally accepted thresholds for key parameters, including: pH (6.5–8.5), TDS (< 1000 mg/L), Turbidity (< 5 NTU), NO₃⁻ (< 50 mg/L), NO₂⁻ (< 3 mg/L), Cl⁻, SO₄^2^⁻ (< 250 mg/L). A compliance analysis was conducted to assess the percentage of samples meeting WHO standards for each parameter. The comparison provides insights into the extent to which water quality in Palestinian areas and Israeli settlements aligns with international health-based standards, and serves as a foundation for assessing environmental risk and infrastructure adequacy within the broader environmental justice framework.

#### Environmental Stress Index (ESI) development

A composite Environmental Stress Index (ESI) was developed to quantify cumulative environmental pressure across the study area. The index integrates multiple normalized indicators representing air quality, water quality, and population density, providing a standardized measure of environmental stress that supports spatial comparison between the Palestinian localities (Dura City and Fawwar Refugee Camp) and the Israeli settlement (Nahal Negohot). The ESI was constructed using a weighted sum of normalized values for the following parameters: Air Quality Index (AQI), Water quality indicators: nitrate (NO₃⁻), total dissolved solids (TDS), electrical conductivity (EC), chloride (Cl⁻), and pH, Population density.

Each parameter was normalized using Min–Max scaling to standardize values across different units and ranges. Parameters negatively associated with environmental quality (e.g., AQI, NO₃⁻, EC) were normalized directly, while parameters with positive environmental implications (e.g., Dissolved Oxygen, if included) were inversely normalized when applicable.

To construct the ESI, each parameter was assigned a relative weight based on its environmental significance, supported by a combination of literature review, expert judgment, and data quality considerations. The following equation represents the sample ESI formulation used in this study:

$${ESI}_{.}={0.25}_{AQInorm }+{0.15}_{\mathrm{NO}3\text{norm }}+{0.1}_{\mathrm{TDSnorm}}+{0.1}_{\mathrm{ECnorm}}+{0.1}_{\mathrm{Clnorm}}+{0.05}_{\mathrm{pHnorm}}+{0.25}_{Popno}$$

To assess the robustness of the index, a sensitivity analysis was conducted by varying the assigned weights and observing their influence on final ESI scores. Results confirmed that AQI and population density had the strongest effect on index variability.

#### Water supply analysis (2014–2024)

This analysis examines water supply disparities between Palestinian areas (Dura City and Fawwar Refugee Camp) and the Israeli settlement (Nahal Negohot) over the period 2014 to 2024. The aim is to assess both the extent and the evolution of inequalities in water access across the study area. Annual data on total water supply and population were collected from municipal sources. Per capita water supply (liters/person/day) was calculated for each locality and year, and classified according to World Health Organization (WHO) guidelines: < 50 L/day: Critical, 50–80 L/day: Poor, 80 L/day: Adequate. The data were organized in an Excel table containing fields for Year, City Code, Total Supply, Population, and Per Capita, and were joined to spatial layers using City Code and Year. Multi-year integration was achieved using relates and time-enabled layers in ArcGIS Pro. A series of temporal maps were generated to visualize annual changes and long-term trends in water supply. These visualizations support the identification of systemic disparities in water service provision and highlight persistent gaps between the Palestinian localities and the Israeli settlement.

#### Hotspot and spatial risk analysis

To identify areas of statistically significant environmental stress, hotspot analysis was conducted using the Getis-Ord Gi* spatial statistic. This method detects spatial clusters where values are significantly higher (hotspots) or lower (cold spots) than the expected local mean. A significance threshold of p < 0.05 was applied to determine meaningful clustering.

The analysis targeted key water quality parameters—nitrate (NO₃⁻), nitrite (NO₂⁻), total dissolved solids (TDS), and electrical conductivity (EC)—using point-based field measurements. The results highlighted zones with concentrated contamination levels that may pose elevated environmental and public health risks.

These hotspots were then spatially overlaid with population density maps and school locations to assess the exposure of vulnerable groups, particularly in densely populated areas with limited infrastructure. The spatial intersection of contamination zones and human activity provided an evidence-based layer for evaluating environmental risk and prioritizing intervention areas within the broader environmental justice framework.

#### Topographic and hydrological analysis for pollution risk zoning

To quantify terrain-induced susceptibility to pollutant transport and to enhance the spatial assessment of environmental stress across the study sites—Dura City, Fawwar Refugee Camp, and the Nahal Negohot settlement—a topographic and hydrological analysis was performed using a high-resolution Digital Elevation Model (DEM). This procedure integrates pollution concentration layers with hydrological flow dynamics to produce a composite Pollution Risk Index (PRI), capturing the joint effects of contaminant severity and terrain-driven flow potential.

##### DEM processing

The DEM was analyzed in ArcGIS Pro to extract key hydrological parameters relevant to surface water movement and subsurface infiltration pathways. This was especially important given that springs and wells in the study area access aquifers situated at depths between 15 and 20 m. The resulting flow accumulation raster was reclassified into five ordinal classes (1 to 5), representing hydrological susceptibility from low (minor flow) to high (major flow paths or basins). This classification serves as a proxy for the landscape’s potential to facilitate pollutant movement.

##### Multi-criteria pollution risk zoning

To integrate pollution severity with hydrological vulnerability, a multi-criterion weighted overlay analysis was conducted using the Raster Calculator in ArcGIS Pro. Three input layers were included: Reclassified IDW Raster of TDS concentrations, Reclassified IDW Raster of Nitrate (NO₃⁻) concentrations, Reclassified Flow Accumulation Raster, representing hydrological influence. Each raster was standardized into five ordinal classes, and weights were assigned based on environmental relevance: TDS: 35% (0.35), Nitrate (NO₃⁻): 35% (0.35), Flow Accumulation: 30% (0.30).

The Pollution Risk Index (PRI) was computed using the following formula:

$${\mathbf{PRI}}\;=\;\left({\mathbf{TDS}}\;\times\;{\boldsymbol{0.35}}\right)\;+\;\left({\mathbf{NO}}_{\boldsymbol{3}{^-}}\;\times\;{\boldsymbol{0.35}}\right)\;+\;\left({\mathbf{Flow Accumulation}}\;\times\;{\boldsymbol{0.30}}\right)$$

Where each term represents the normalized and reclassified value of its respective parameter. The final output is a continuous raster, which was subsequently reclassified into five discrete risk categories, ranging from 1 (very low risk) to 5 (very high risk).

This composite index enables the identification of spatial hotspots where pollutant concentrations overlap with terrain features conducive to pollutant transport. It serves as a critical input for downstream analysis and supports risk-based prioritization of monitoring and mitigation efforts.

##### Future scenarios and forecasting

Scenario Modeling: Simulated increased population and pollution levels, Recalculated ESI for future years, Identified potential high-risk areas.

Interpretation: Results used for urban planning and prioritization of interventions.

## Results

### Water quality assessment

**Fig. 2:**
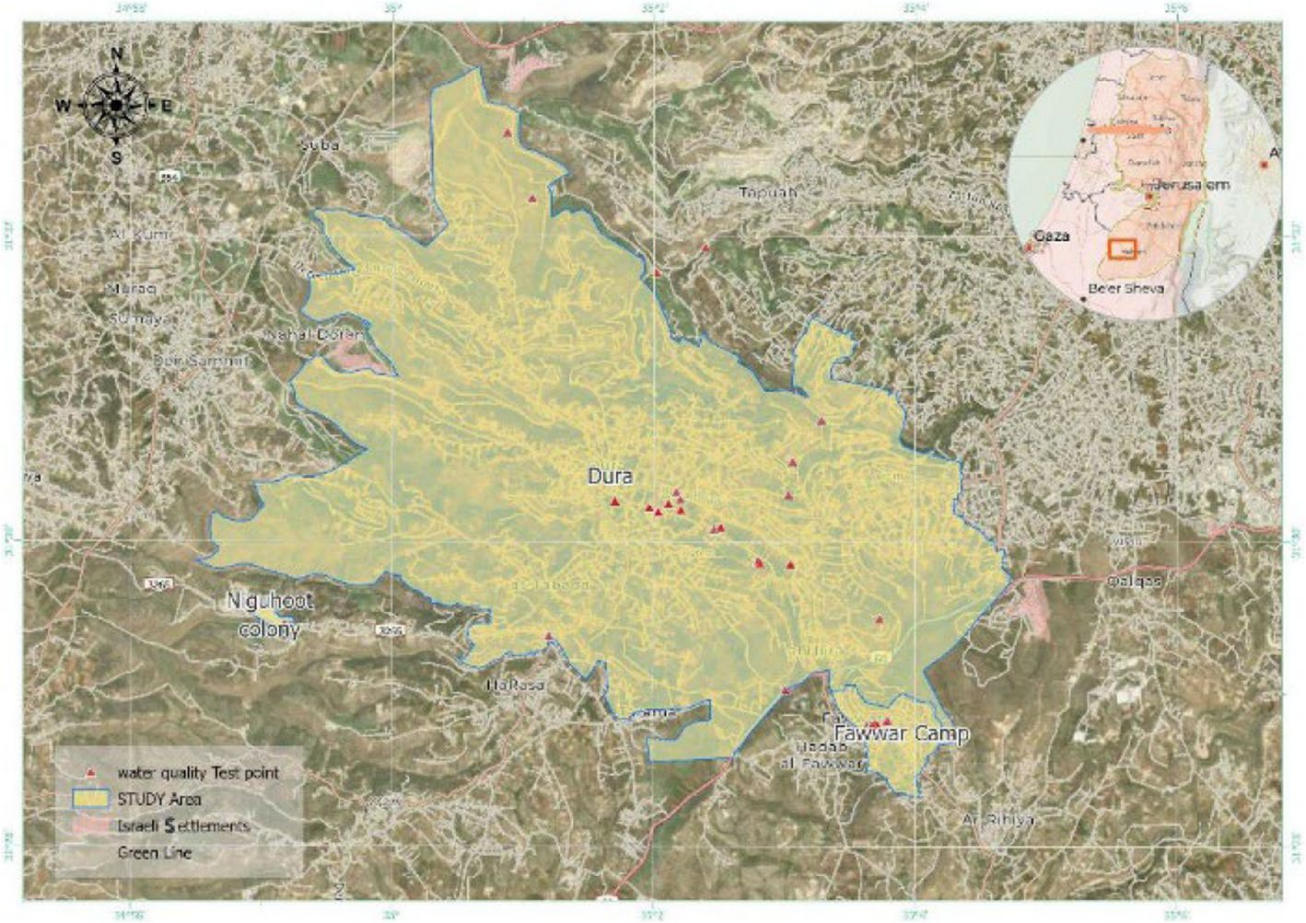
Map of sampling locations in the study area (Dura City, Fawwar Refugee Camp, and Nahal Negohot Settlement)

Water quality across the three localities—Dura City, Al-Fawwar Refugee Camp, and the Nahal Negohot settlement—was evaluated through analysis of key physicochemical parameters, including nitrate (NO₃⁻), nitrite (NO₂⁻), total dissolved solids (TDS), electrical conductivity (EC), chloride (Cl⁻), and pH. Mean concentrations for each parameter were calculated and compared with World Health Organization (WHO) drinking water standards to identify exceedances. These parameters were selected due to their relevance to groundwater quality, public health, and domestic water usability. Figure 2 illustrates the spatial distribution of sampling locations across the study area.

The comparative water quality parameters for Dura City, Fawwar Camp, and Nahal Negohot are summarized in Table 1. The table presents minimum, mean, and maximum values for key indicators, including nitrate (NO₃⁻), nitrite (NO₂⁻), total dissolved solids (TDS), electrical conductivity (EC), pH, chloride (Cl⁻), phosphate (PO₄3⁻), and turbidity. These values are compared against the World Health Organization (WHO) and United States Environmental Protection Agency (EPA) guideline limits to assess compliance with international standards. The data reveal notable variations among the three locations, with some parameters exceeding recommended limits, highlighting potential risks to public health and the need for targeted water quality management.

The measured nitrate (NO₃⁻) concentration was 49 mg/L, slightly exceeding the U.S. EPA guideline of 44.3 mg/L while remaining within the WHO guideline limit of 50 mg/L. The recorded pH value was 7.22, which falls within the WHO-recommended range of 6.5–8.5. Electrical conductivity (EC) was 912 µS/cm, substantially below the WHO reference value of 2,500 µS/cm. Total dissolved solids (TDS) were measured at 529 mg/L, exceeding the EPA guideline of 500 mg/L but remaining below the WHO recommended limit of 600 mg/L (Table 1).

**Table 1:** presents the comparative water quality parameters measured in Dura City, Fawwar Camp, and Nahal Negohot, alongside WHO and EPA guideline values

Parameter	WHO Limit	EPA Limit	Dura City (Min–Mean–Max)	Fawwar Camp (Min–Mean–Max)	Nahal Negohot (Single/Range)
Nitrate (NO₃⁻) [mg/L]	50	44.3	0–254.5–1,700	51.5–85.3–147.1	49
Nitrite (NO₂⁻) [mg/L]	3	–	0–12–28	1–6 – 9	0.8
Total Dissolved Solids (TDS) [mg/L]	600	500	400–1,700–2,100	188–436–724	529
Electrical Conductivity (EC) [µS/cm]	2,500	–	500–1,600–2,600	750–1,100–1,500	912
pH	6.5–8.5	6.5–8.5	6.5–7.8–8.6	6.94–7.55–8.07	7.22
Chloride (Cl⁻) [mg/L]	250	–	120–245–332	100–210–300	45
Phosphate (PO₄^3^⁻) [mg/L]	0.1	–	0.02–0.78–1.24	0.01–0.35–0.60	0.05
Turbidity [NTU]	1 (guideline)	5 (relaxed)	0.3–2.1–5.69	0.5–1.8–5.69	0.4

Elevated concentrations of nitrates, nitrites, and phosphates are widely recognized as strong indicators of contamination of drinking water by wastewater or sewage, particularly in areas relying on cesspits or lacking adequate sanitation infrastructure.

**Fig. 3:**
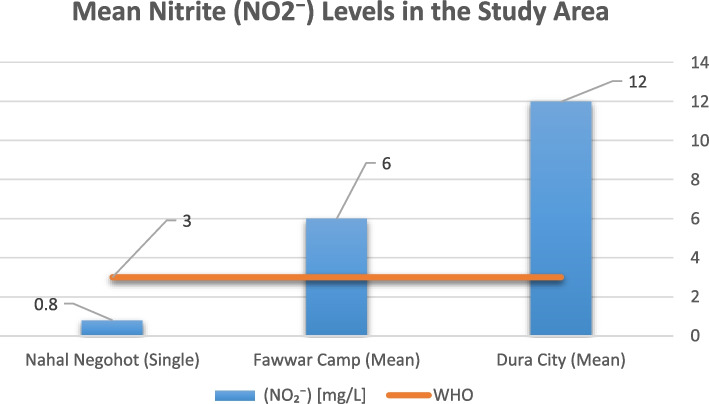
Mean Nitrite (NO_2_⁻) levels in the study area

Analysis of nitrogen compounds revealed distinct spatial patterns. Nitrite (NO₂⁻) concentrations, shown in Fig. 3, exceeded the WHO guideline value of 3 mg/L in multiple samples from Dura City and Al-Fawwar Refugee Camp. In contrast, nitrite concentrations in Nahal Negohot remained consistently below 1 mg/L and within WHO limits.

**Fig. 4:**
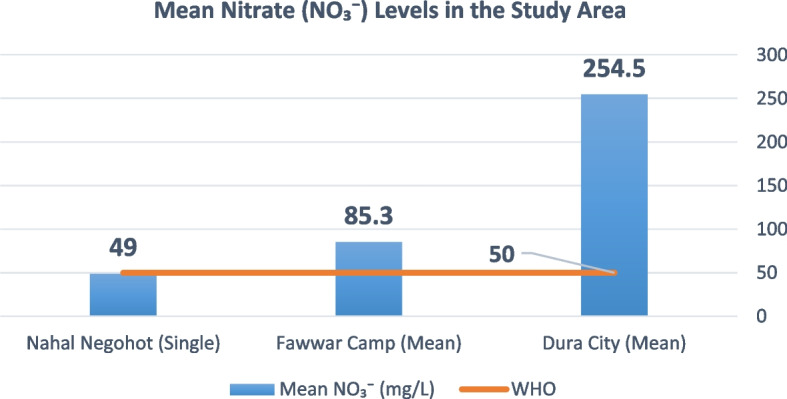
Mean Nitrate (NO₃⁻) levels in the study area

Nitrate (NO₃⁻) concentrations exhibited pronounced variability across the study area Fig. 4. In Dura City, nitrate levels frequently exceeded the WHO guideline of 50 mg/L, with a mean concentration of approximately 254.5 mg/L and maximum values reaching up to 1,667.5 mg/L in some samples. Al-Fawwar Refugee Camp showed moderate exceedances, with a mean nitrate concentration of approximately 95.8 mg/L and maximum values of 147.1 mg/L. In contrast, nitrate concentrations in Nahal Negohot remained below the WHO guideline across all sampled locations.

**Fig. 5:**
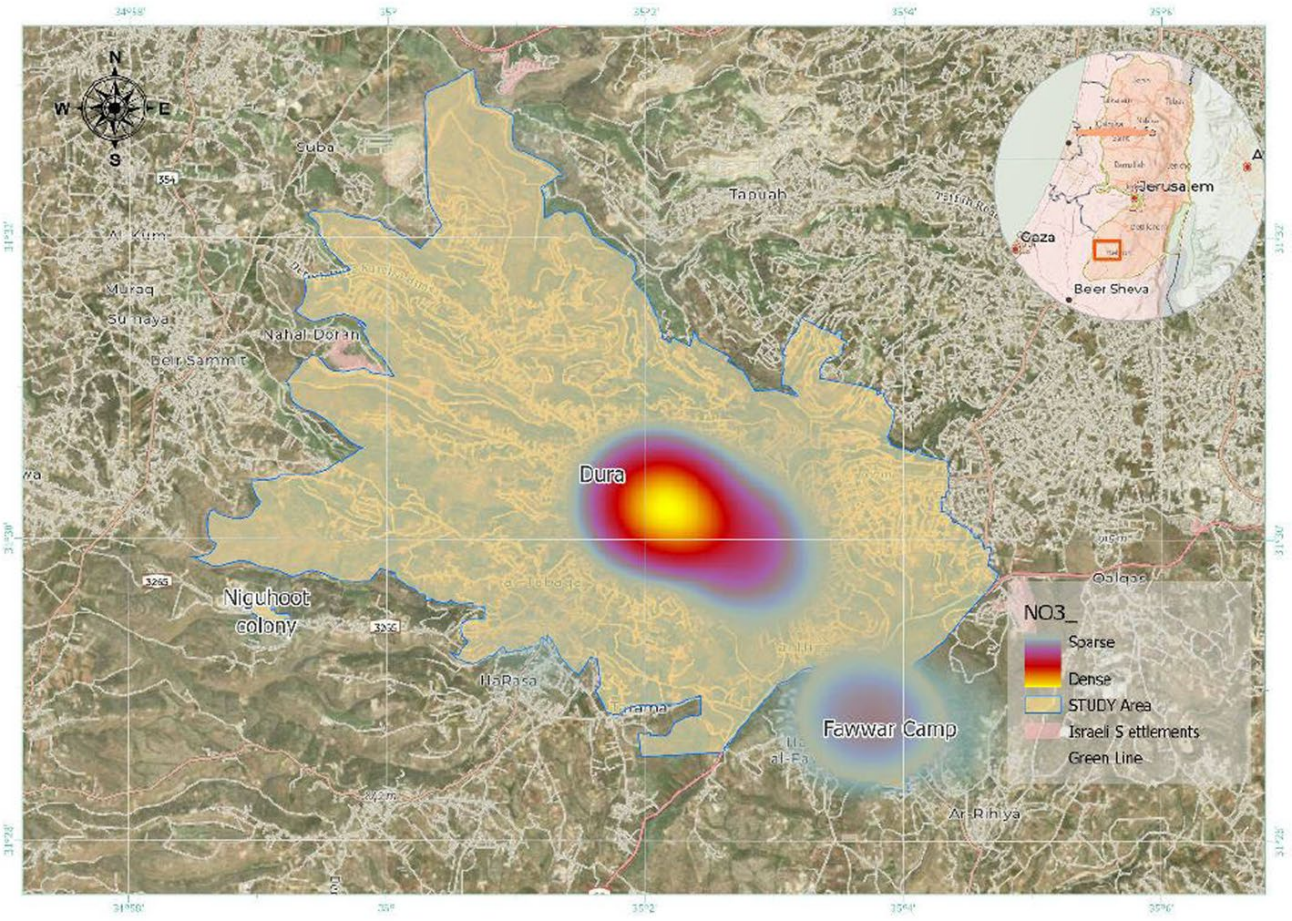
Map of Nitrate (NO₃⁻) distribution in the study area with heat map

Figure 5 also presents a nitrate density heat map, illustrating areas of high and low nitrate concentration. High-density zones (red and yellow) are concentrated mainly in central Dura City, while lower-density zones (green and blue) are observed in areas surrounding Nahal Negohot. Al-Fawwar Refugee Camp displays intermediate nitrate density levels.

**Fig. 6:**
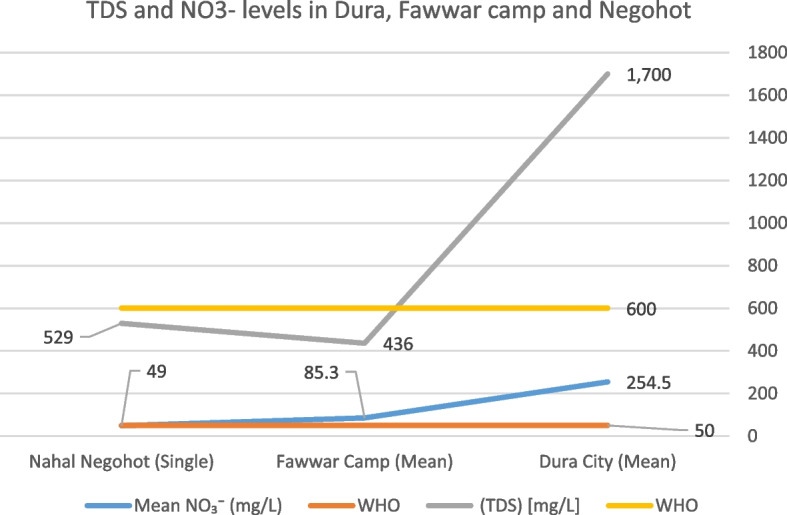
Map of comparison of Total Dissolved Solids (TDS) and Nitrate (NO₃⁻) concentrations across Dura City, Fawwar Refugee Camp, and Nahal Negohot Settlement

Figure 6 compares total dissolved solids (TDS) and nitrate (NO₃⁻) concentrations across the study area using bar representations. Samples from Dura City and Al-Fawwar Refugee Camp show elevated TDS levels, reaching up to approximately 1,000 mg/L and exceeding the WHO desirable limit of 600 mg/L. In contrast, TDS concentrations in Nahal Negohot remain below 600 mg/L. Nitrate concentrations follow a similar spatial pattern.

**Fig. 7:**
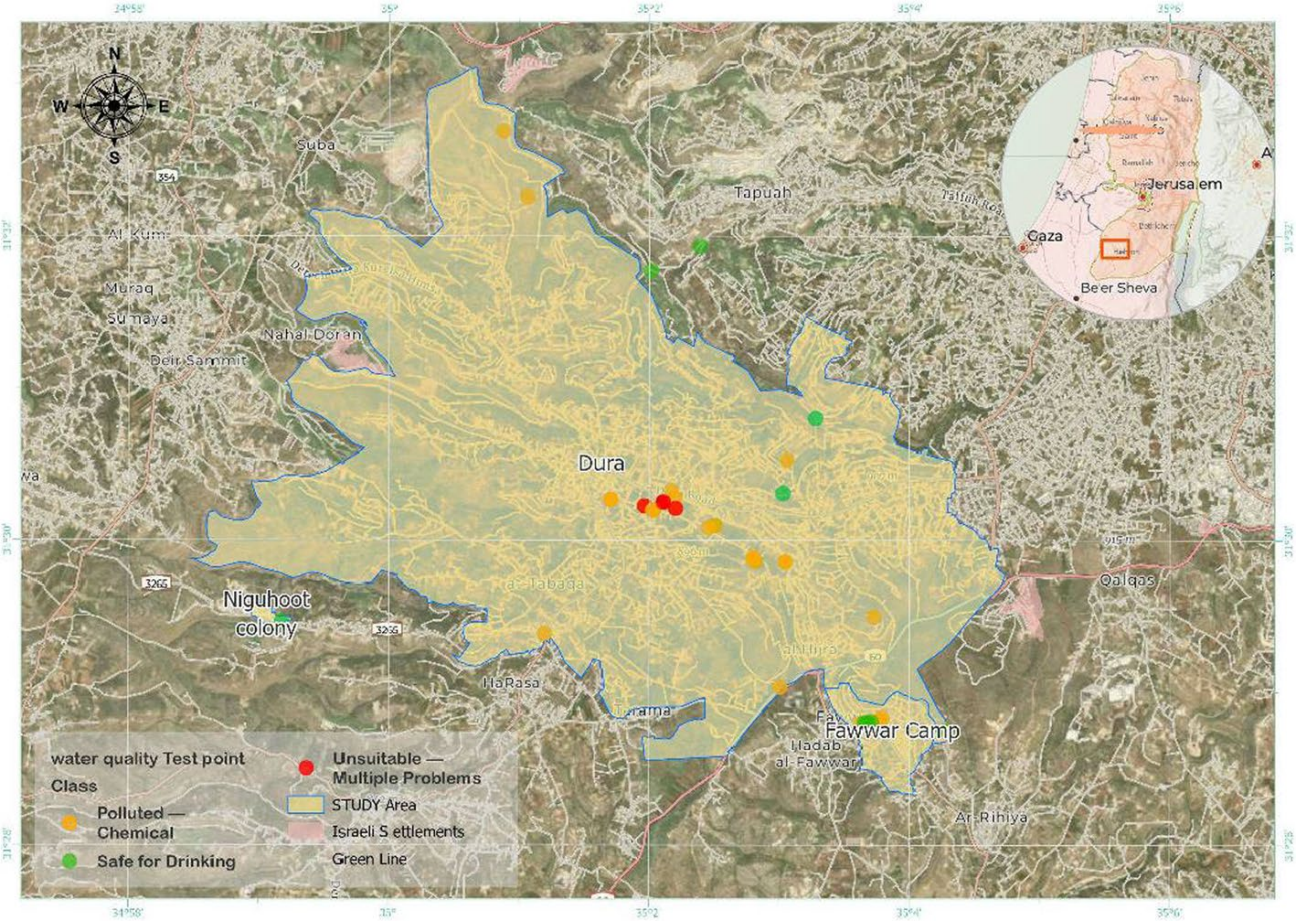
Final classification of water samples based on physicochemical parameter analysis and WHO drinking water standards

The spatial classification of water quality status across the study area is presented in Fig. 7, categorizing samples according to WHO drinking water standards into three classes: safe for drinking**,** chemically polluted**,** and unsuitable due to multiple exceedances. Most samples from Dura City and Al-Fawwar Refugee Camp fall within the chemically polluted or unsuitable categories, while the sample from Nahal Negohot is classified as safe for drinking.

### Spatial interpolation of water quality parameters using Inverse Distance Weighting (IDW)

**Fig. 8:**
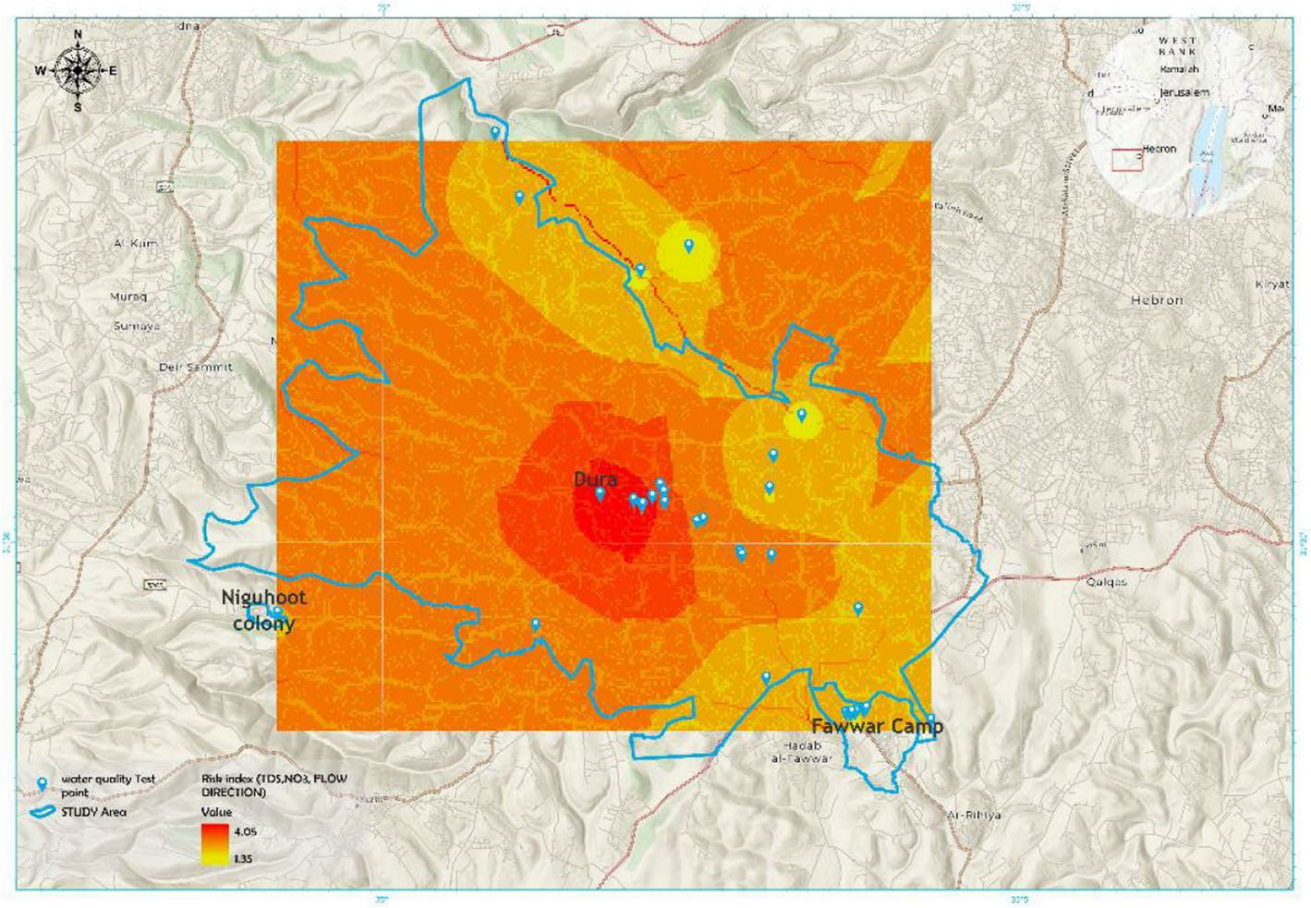
Spatial Pollution Risk Index (PRI) derived from TDS, Nitrate, and Flow Accumulation Analysis

To assess the spatial distribution of groundwater quality across the study area, the Inverse Distance Weighting (IDW) interpolation technique was applied using ArcGIS. Continuous spatial layers were generated for key hydro chemical parameters—including nitrate (NO₃⁻), total dissolved solids (TDS), and electrical conductivity (EC)—in addition to a composite Pollution Risk Index (PRI). The PRI was developed through hydrological modeling incorporating flow direction and flow accumulation to capture spatial pollution dynamics (Fig. 8).

The IDW interpolation of nitrate concentrations reveals pronounced spatial variability across the study area. Dura City exhibits the highest nitrate concentrations, exceeding 1,500 mg/L in several locations and far surpassing the WHO guideline value of 50 mg/L. Al-Fawwar Refugee Camp shows nitrate concentrations ranging between 50 and 300 mg/L. In contrast, nitrate concentrations in Negohot settlement consistently remain below 50 mg/L.

Spatial interpolation of TDS indicates that Dura City records the highest concentrations, with values exceeding 1,700 mg/L compared to the WHO guideline of 600 mg/L. In Al-Fawwar Refugee Camp, TDS concentrations range between 400 and 900 mg/L. Negohot settlement exhibits the lowest TDS values, generally below 250 mg/L.

The EC interpolation map Fig. 10 shows that Dura City has the highest conductivity values, exceeding 2,600 µS/cm and surpassing the WHO threshold of 2,500 µS/cm. Al-Fawwar Refugee Camp displays moderate EC values ranging from approximately 750 to 1,500 µS/cm. Negohot settlement again shows the lowest EC values, consistently below 500 µS/cm.

The Pollution Risk Index (PRI), presented in Fig. 11, was generated using a weighted overlay of standardized raster layers: TDS (35%), nitrate (NO₃⁻) (35%), and flow accumulation (30%). The resulting PRI map reveals clear spatial differentiation in pollution vulnerability. Dura City records the highest PRI values, reaching up to 4.05. Al-Fawwar Refugee Camp shows moderate PRI values ranging between 2.0 and 3.5. Negohot settlement presents the lowest PRI values, between 1.35 and 2.0.

### Analysis of water quality parameters in

#### Dura City

Groundwater quality in Dura City was assessed using key chemical and physical parameters, including nitrate (NO₃⁻), pH, electrical conductivity (EC), total dissolved solids (TDS), chloride (Cl⁻), phosphate (PO₄3⁻), and turbidity, and compared against WHO and EPA standards. Nitrate (NO₃⁻) concentrations exhibited substantial spatial variability, ranging from near-zero values to extreme contamination hotspots. Exceptionally high nitrate levels were recorded in several samples, including 600 mg/L, 300 mg/L, 1,300 mg/L, 1,500 mg/L, and 1,700 mg/L. These values exceed the WHO guideline limit of 50 mg/L by approximately 6 to 34 times, indicating severe localized contamination. Measured pH values ranged between 6.5 and 8.6. Most samples were neutral to mildly alkaline (7.8–8.5), with Sample 18 slightly exceeding the WHO upper guideline value (8.5). Electrical conductivity (EC) values varied from 500 to 2,000 µS/cm, with several samples approaching the WHO recommended threshold of 2,500 µS/cm, indicating elevated dissolved ionic content Table 1.

**Fig. 9:**
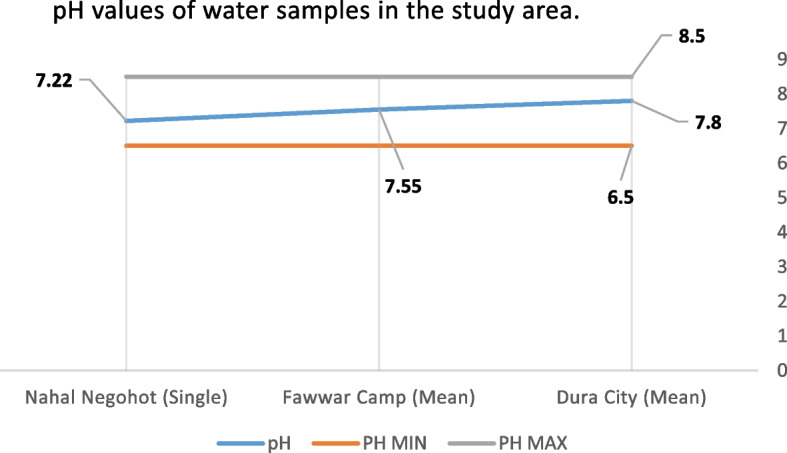
pH measurements for water samples across the study area

Total dissolved solids (TDS) concentrations were notably high in multiple locations, reaching 2,000–2,100 mg/L in contamination hotspots. These values significantly exceeded both WHO (600 mg/L) and EPA (500 mg/L) guideline limits. Chloride (Cl⁻) concentrations surpassed the recommended limit of 250 mg/L in several samples, including 301.3 mg/L and 332.6 mg/L. Phosphate (PO₄3⁻) levels exceeded the 0.1 mg/L guideline in multiple samples, with maximum concentrations reaching 1.24 mg/L. Turbidity values exceeded the recommended threshold of 1 NTU in selected samples, ranging between 5.56 and 5.69 NTU Table 1. Based on compliance with international drinking water standards, groundwater samples were classified into three categories: Safe for Drinking**,** polluted — Chemical**,** and Unsuitable — Multiple Problems**,** revealing pronounced spatial variability in groundwater quality across Dura City Fig. 9.

#### Al-Fawwar refugee camp

**Fig. 10:**
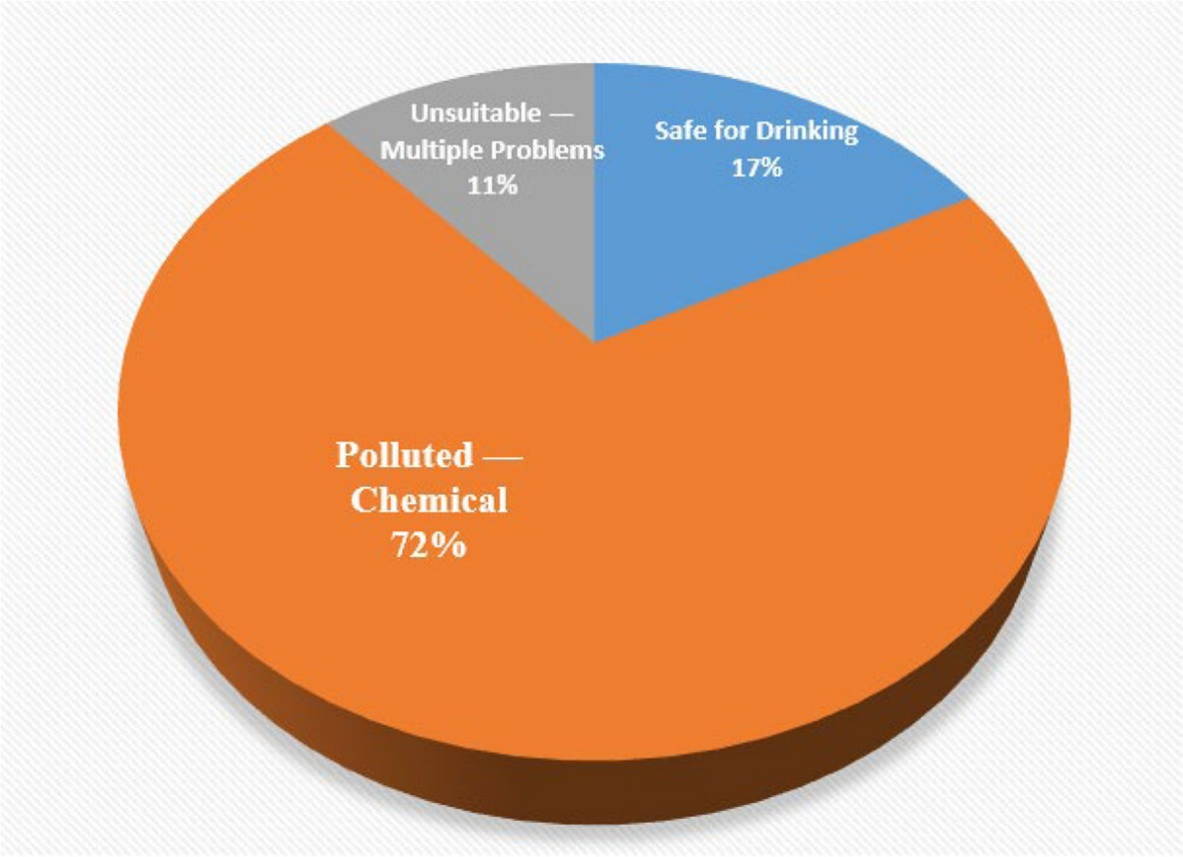
Classification of water samples in Dura City

Groundwater quality in Al-Fawwar Refugee Camp was evaluated based on key chemical and physical parameters, including nitrate (NO₃⁻), pH, total dissolved solids (TDS), and turbidity, and compared against WHO and EPA drinking water standards. Nitrate (NO₃⁻) concentrations were measured in 19 groundwater samples. The results indicate that 58% of the samples exceeded both the WHO guideline (50 mg/L) and the EPA standard (44.3 mg/L). Nitrate concentrations ranged from 51.5 to 147.1 mg/L, with a mean value of 85.26 mg/L. On average, nitrate levels were approximately 1.71 times higher than the WHO guideline and 1.93 times higher than the EPA limit. Measured pH values ranged from 6.94 to 8.07, with a mean of 7.55. All samples fell within the WHO-recommended pH range of 6.5–8.5. Total dissolved solids (TDS) concentrations varied between 188 and 724 mg/L. Approximately 58% of the samples exceeded the EPA guideline of 500 mg/L, while 37% surpassed the WHO guideline of 600 mg/L. Turbidity values exceeded the WHO recommended limit of 1 NTU in two samples. These values also slightly exceeded the WHO’s relaxed turbidity threshold of 5 NTU Table 1. Following assessment of groundwater quality parameters—including nitrate (NO₃⁻), pH, electrical conductivity (EC), total dissolved solids (TDS), chloride (Cl⁻), phosphate (PO₄3⁻), and turbidity—each sample was classified according to its compliance with international standards. Based on this classification, groundwater samples were categorized into two groups: potable and chemically contaminated. The spatial distribution of these categories is illustrated in Fig. 10.

#### Nahal Negohot

Groundwater quality in Nahal Negohot was assessed using a single water sample supplied by Mekerot, the Israeli national water company. The analysis was limited to this single available sample due to restricted access for Palestinian researchers. Accordingly, the findings should be interpreted as indicative rather than representative of overall groundwater conditions in the area.

**Fig. 11:**
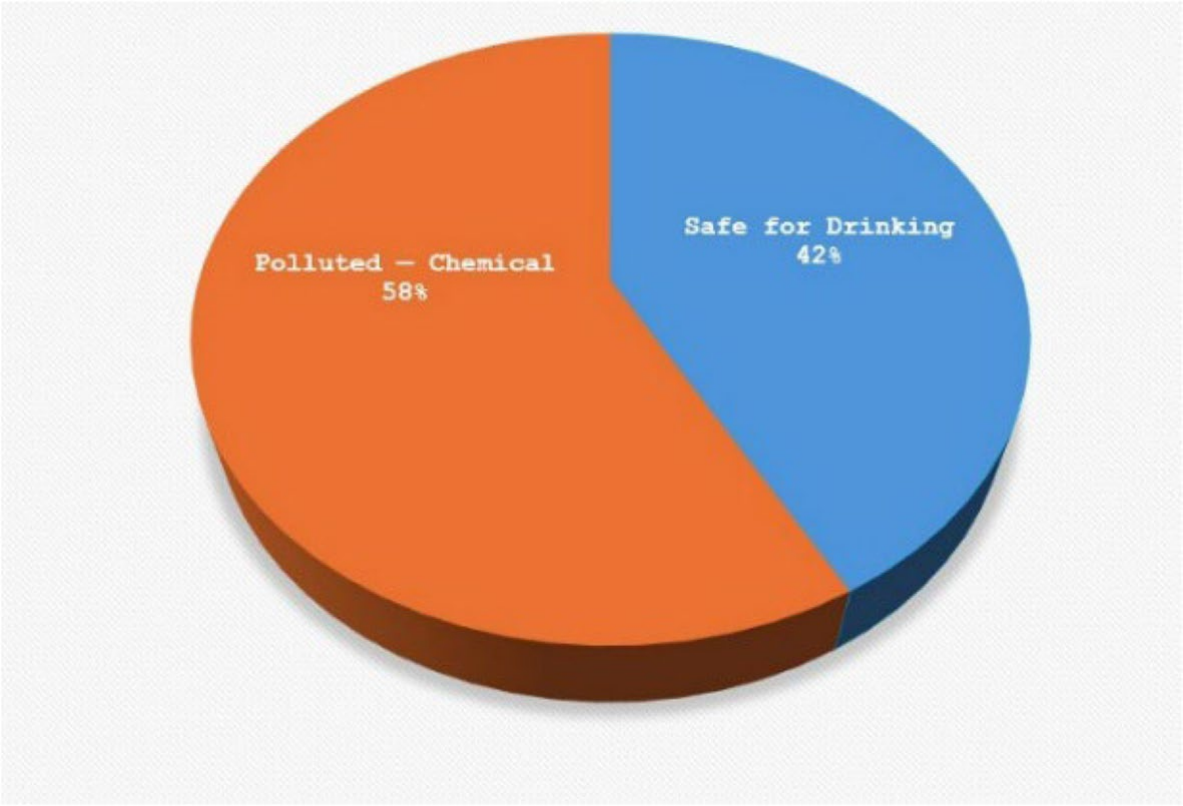
Classification of water samples in Al Fawwar refugee camp

Given the reliance on a single sample, these results provide a descriptive snapshot of selected physicochemical parameters at the time of sampling and do not allow for statistical generalization or temporal trend analysis. Within these constraints, the measured values suggest partial compliance with international drinking water standards for the sampled source. These data have been visually represented in a Fig. 11, which was constructed based on the measured parameters and in accordance with WHO standards for classifying physicochemical water quality indicators.

#### Population vs. water allocation disparity (2024)

**Fig. 12:**
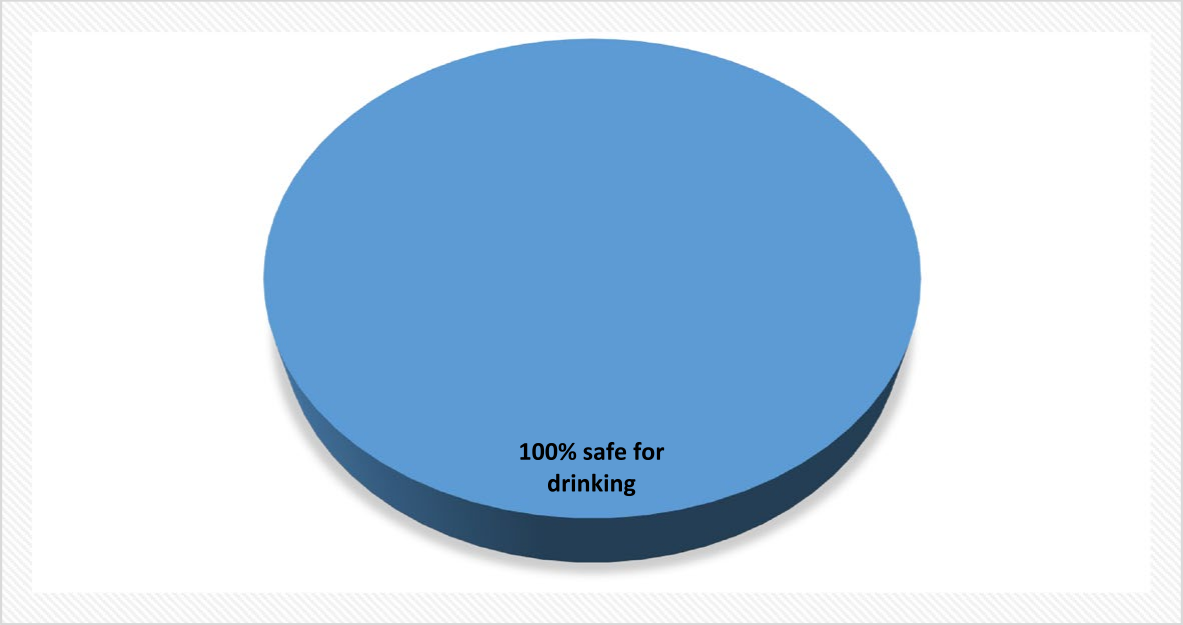
Classification of groundwater quality parameters in Nahal Negohot based on a single water sample

To assess the relationship between demographic demand and water allocation, Fig. 12 compares population size with the share of total water supply for each locality in 2024. Dura City, with an estimated population of approximately 47,780 residents, received 13.5% of the total water distributed among the three localities. Al-Fawwar Refugee Camp, with a population of around 9,056, received 7.2% of the total water supply. In contrast, the Israeli settlement of Nahal Negohot, with a population of only 425 residents, received 79.3% of the total water supplied in 2024. The right panel of Fig. 12 presents per capita water supply levels for the same year. Average daily per capita water supply was estimated at 64.6 L per day in Dura, 53.0 L per day in Al-Fawwar Camp, and 650.0 L per day in Nahal Negohot. These results demonstrate substantial variation in both total and per capita water allocation among the three localities.

#### Environmental Stress Index (ESI) analysis

**Fig. 13:**
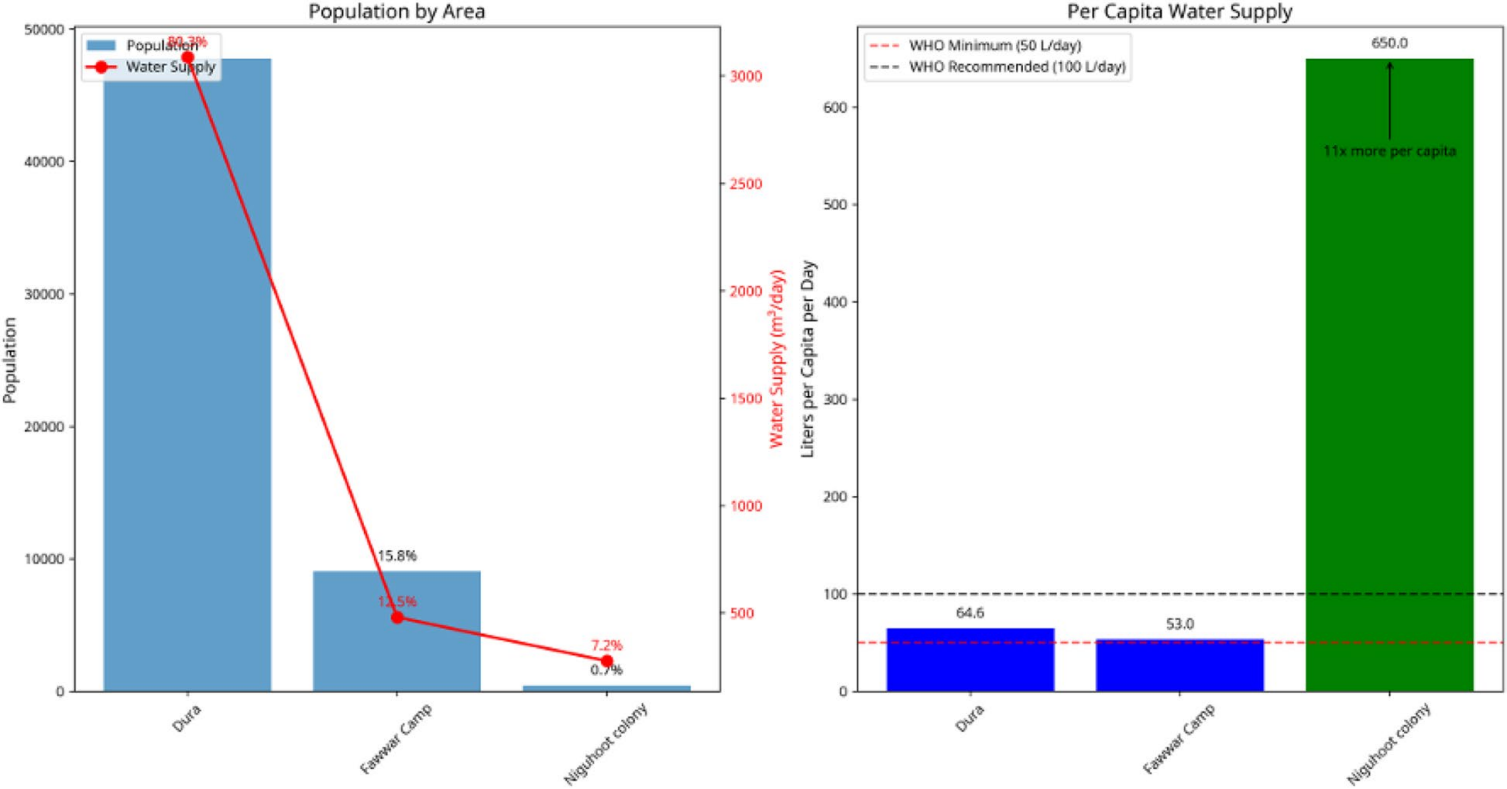
(Left) population vs. share of water supply, and (Right) per capita water supply in 2024

**Fig. 14:**
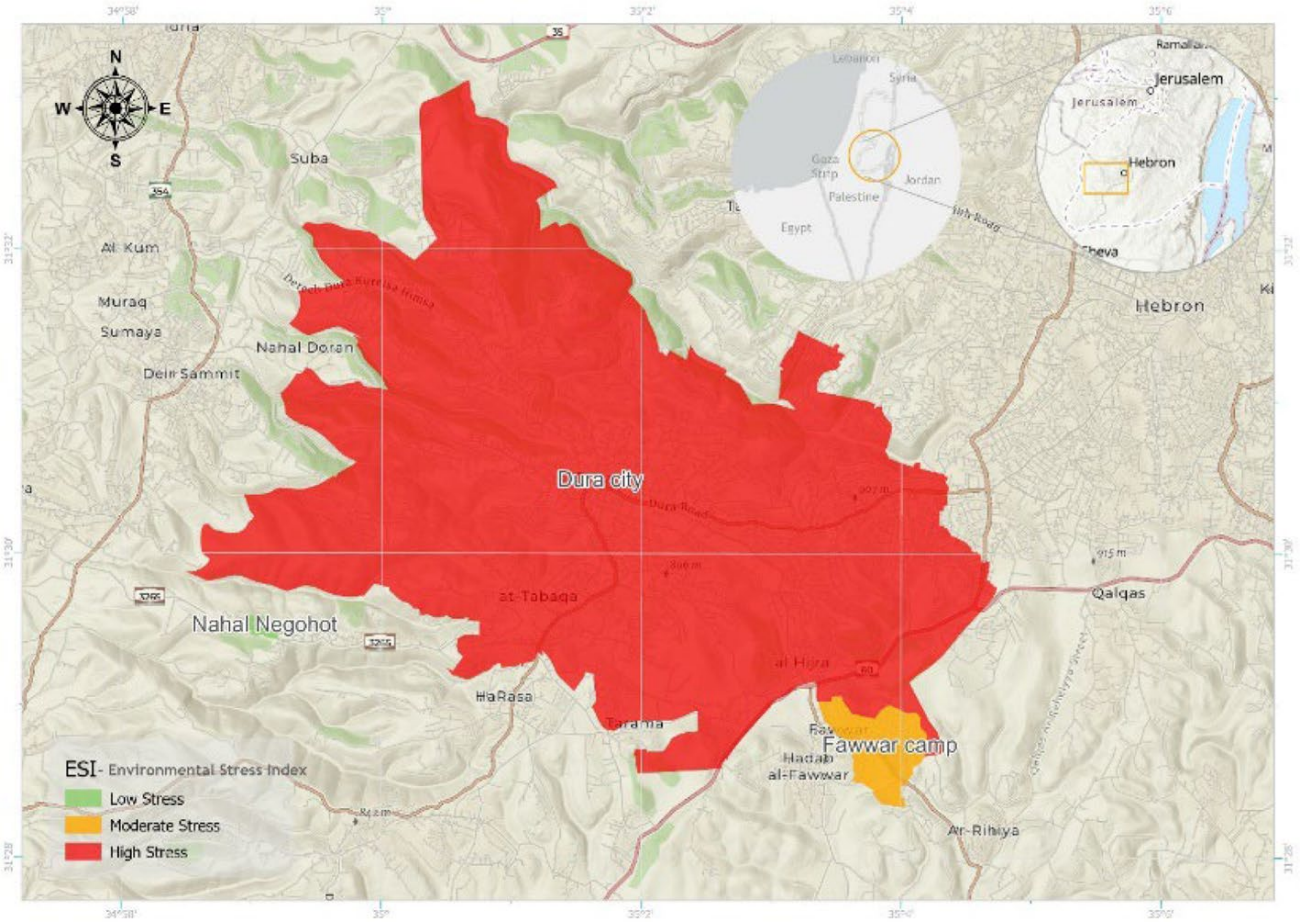
Environmental Stress Index (ESI) distribution in the study area, 2024

The Environmental Stress Index (ESI) analysis integrates multiple environmental variables—including water quality, wastewater management, air quality, and land use patterns—into a composite index designed to assess overall environmental stress across the study area. The index was calculated using the methodology outlined in this study, based on data aggregated for the period 2019–2024 and processed through Geographic Information Systems (GIS). Figure 13 presents the spatial distribution of the ESI, classifying environmental stress into three categories: Low Stress (green)**,** Moderate Stress (yellow)**,** and High Stress (red). The map provides a comparative visualization of cumulative environmental pressures resulting from factors such as untreated sewage, waste burning, and limited water availability. The results indicate that Dura City is predominantly classified under High Environmental Stress (red). Al-Fawwar Refugee Camp is largely categorized as Moderate Environmental Stress (yellow)**.** In contrast, the Israeli settlement of Nahal Negohot is classified as Low Environmental Stress (green). Peripheral areas located farther from major pollution sources also exhibit Low Stress levels. These classifications reveal clear spatial contrasts in cumulative environmental stress among the three localities (Fig. 14).

## Discussion

The findings of this study are strongly consistent with, and extend, existing literature on groundwater quality, infrastructure disparities, and environmental justice in the West Bank. The elevated concentrations of nitrate, TDS, EC, and chloride observed in Dura City and Al-Fawwar Refugee Camp corroborate previous assessments identifying untreated wastewater infiltration, agricultural runoff, and excessive groundwater abstraction as dominant contamination pathways in Palestinian localities [5, 12]. However, the spatially explicit comparison with Nahal Negohot demonstrates that groundwater degradation is not solely a function of hydrogeological sensitivity or local land use, but is critically shaped by differential access to water infrastructure, treatment systems, and regulated supply.

The consistently low contaminant levels and reduced pollution risk indices recorded in Nahal Negohot settlement align with studies emphasizing the protective role of centralized wastewater networks, systematic monitoring, and institutional capacity in mitigating water quality deterioration [7, 9]. In contrast, the fragmented infrastructure, high non-revenue water, reliance on cesspits and septic systems, and occasional poor management in Palestinian areas—widely documented in prior research [1, 2, 23]—are directly reflected in the elevated pollution and environmental stress indices identified in this study. These results provide empirical spatial evidence that infrastructure deficits translate into cumulative chemical exposure and heightened public health risk.

From an environmental justice perspective, the inverse relationship between population size and water allocation, coupled with stark disparities in groundwater quality, reinforces arguments that water access and environmental risk in the West Bank are structured by distributive, procedural, and recognition injustices rather than demographic need or technical efficiency [18, 19, 23]. The Environmental Stress Index further illustrates how inadequate water supply, untreated wastewater, and limited environmental management converge spatially in Palestinian localities, producing chronic exposure to environmental hazards. These patterns are consistent with conflict-context literature demonstrating how restrictions under the Oslo II Agreement and Area C designation constrain Palestinian capacity to develop sanitation and water treatment infrastructure, thereby institutionalizing environmental vulnerability [23, 25].

By integrating spatial water quality analysis with composite risk indices, this study advances the literature by quantitatively demonstrating how political and infrastructural asymmetries manifest as place-based environmental stress. The findings thus move beyond descriptive accounts of water scarcity and contamination to provide measurable evidence that groundwater degradation in Palestinian urban and refugee settings is structurally produced, reinforcing broader patterns of environmental injustice and undermining progress toward equitable water governance and Sustainable Development Goal 6.

Despite the robustness of the spatial analysis and the integration of multiple water quality and environmental stress indicators, this study is subject to certain limitations. Groundwater sampling was constrained by accessibility and temporal coverage, which may not fully capture seasonal variability in contamination levels. Additionally, the reliance on available institutional data for water allocation may underestimate informal or unreported extraction. These limitations do not undermine the overall findings but highlight the need for longitudinal monitoring and expanded sampling frameworks.

The discussion highlights that addressing groundwater degradation and environmental stress in Palestinian localities requires more than technical remediation. The findings underscore the necessity of integrated sanitation infrastructure, enhanced monitoring systems, and policy interventions that confront the structural and governance-related drivers of environmental inequality. These implications provide a critical foundation for the conclusions and recommendations presented in the following section.

## Limitations and path forward

This study faces several limitations that should be acknowledged to contextualize its findings and guide future research and interventions. First, data gaps in official statistics and environmental records, particularly in areas such as Israeli settlements, may affect the accuracy of the analysis. For example, precise population figures for Nahal Negohot rely on approximate estimates due to the absence of publicly accessible official data. The spatial resolution of available datasets is also limited, which may obscure localized environmental variations and potentially overlook micro-scale differences in exposure or vulnerability. In addition, inconsistencies in temporal coverage across datasets restrict comprehensive analysis of long-term trends, making it challenging to assess changes in environmental quality and population pressures over the study period. Observed patterns alone do not suffice to establish causal links between variables, and further investigation is required to determine the drivers of environmental disparities. Moreover, water quality sampling was disproportionate, with 38 points in Palestinian localities compared to a single representative sample in Israeli settlements, reflecting the centralized water management in the settlements where alternative sources such as contaminated springs or private wells are largely absent. Consequently, the limited availability of independent water sources constrained the scope of sampling and the robustness of comparative analysis.

Within these constraints, the findings indicate an urgent need for coordinated, multi-level interventions that integrate technical solutions, institutional reform, and international engagement. Addressing groundwater contamination and environmental stress in Palestinian localities requires the establishment of decentralized or semi-centralized wastewater treatment facilities where full-scale plants are not immediately feasible, alongside the rehabilitation and sealing of existing septic tanks and cesspits. Such interventions primarily fall under the responsibility of Palestinian local authorities, in coordination with the Palestinian Water Authority and the Ministry of Local Government, supported technically by local universities and research centers, and phased implementation could be facilitated by international donors including the World Bank, KfW, and UNDP. The study also highlights the severe imbalance in per capita water allocation, emphasizing the need for equitable water distribution mechanisms that align with WHO minimum standards for domestic water supply. Policy-level engagement is essential to prioritize underserved Palestinian localities in water allocation planning and to reduce dependency on contaminated local sources.

The spatial variability of contamination and elevated Pollution Risk Index values in Palestinian areas further underscore the necessity for continuous water quality monitoring and dynamic risk assessment. Establishing a standardized monitoring network that integrates routine sampling, GIS-based spatial analysis, and periodic updates of the Environmental Stress Index would enable early detection of emerging hotspots and more efficient targeting of interventions. This function could be led by the Palestinian Water Authority and Environmental Quality Authority in collaboration with academic institutions, while international organizations such as WHO and UNICEF provide methodological guidance, laboratory capacity building, and data harmonization support. Improving water quality outcomes also depends on community engagement and behavioral change, particularly in contexts where infrastructure deficits persist. Awareness programs focusing on water conservation, safe wastewater disposal, and pollution prevention can reduce localized contamination and enhance community resilience, and such initiatives are most effectively implemented through municipalities, local NGOs, schools, and community-based organizations with technical support from international NGOs and UN agencies experienced in WASH programming.

Finally, the structural nature of environmental inequality highlighted in this study necessitates sustained international support that goes beyond project-based funding. International stakeholders, including UN agencies, donor governments, development banks, and environmental justice networks, have a critical role in providing long-term technical assistance, financing, and policy advocacy to address the systemic drivers of unequal environmental governance. Aligning interventions with SDG 6 (Clean Water and Sanitation) and SDG 10 (Reduced Inequalities) can help frame water and environmental justice in Palestine as both a development and a rights-based issue, ensuring that future strategies are comprehensive, sustainable, and equitable.

## Conclusions

This study suggests significant environmental justice disparities in the southern West Bank. Palestinian localities, particularly Dura City and Fawwar Refugee Camp, exhibit severe groundwater contamination, inadequate wastewater management, and limited water supply, whereas the Israeli settlement of Nahal Negohot maintains comparatively high-water quality and low environmental stress. Elevated nitrate and TDS levels in Palestinian areas exceed WHO standards, suggesting the influence of unregulated wastewater discharge, agricultural runoff, and limited infrastructure capacity.

Socioeconomic factors play a pivotal role in shaping water consumption patterns in the Palestinian territories. Unlike norms driven by water abundance, Palestinian consumption is characterized by a focus on meeting basic household needs above all other uses. These conditions, combined with limited incomes, have led to an "essential" and supply-driven consumption pattern. It focuses on drinking and sanitary uses and is almost devoid of recreational uses or irrigation of private green spaces, which constitute a significant portion of consumption in communities with water abundance. Consequently, the low per capita consumption rate does not reflect a societal choice as much as it is a natural outcome of a complex context that imposes an economy of scarcity and reinforces the priority of necessity over luxury.

The Environmental Stress Index confirms that Dura experiences high environmental stress, Fawwar moderate stress, and Nahal Negohot low stress. This indicates that limited capabilities and occasional mismanagement in water and infrastructure development may exacerbate environmental vulnerability in Palestinian communities. These findings suggest that governance, access to infrastructure, and resource allocation are closely linked to environmental risk and public health outcomes.

Overall, the study indicates that effective mitigation of groundwater contamination and environmental stress in the region may require an integrated approach combining technical interventions with administrative reforms, increased capabilities, and international support. Strengthened local governance and community engagement are also essential to address systemic inequalities and promote sustainable water management, ultimately reducing environmental health disparities and advancing environmental justice.

## Data Availability

Data Availability Statement The datasets generated and/or analyzed during the current study are available from the corresponding author, Nidal Nassar, upon reasonable request. Data for this study were collected from multiple sources: The Palestinian Ministry of Health Dura Municipality The Palestinian Water and Environmental Authority Laboratory analyses conducted by the corresponding author Previously published studies and reports All data were processed and analyzed in accordance with ethical and legal regulations applicable to environmental research in Palestine.

## References

[1] Abdullah Murar, Ahmad Sadaqa, Khalid Rabayah, Subhi Samhan, Abdelrahman Tamimi, Walid Sabbah, Ihab Barghothi. The efficiency and institutional performance of the Palestinian water service providers. American Journal of Environmental and Resource Economics. 2017;2(5):162–174. http://www.sciencepublishinggroup.com/j/ajeredio:10.11648/j.ajere.20170205.13.

[2] Abdullah Murar, Ibrahim Awad, Abdel Fattah thasan, Eyad Yagob, Ihab Barghothi, Ahmad Sadaqa, Subhi Samhan, Abdelrahman Tamimi. The impact of water price on the financial sustainability of the Palestinian water service providers. J Environ Prot. 2017;8:1490–1508. http://www.scirp.org/journal/Jeb.

[3] Abu Zarifa SS, Mogheir YK. Gaza City Water Network Operation and Management at Emergency Cases (Unpublished master's thesis). Islamic University of Gaza. 2016. Retrieved from http://search.mandumah.com/Record/734988

[4] Al-Edie, Abeer Mohammad, & Qtaishat, Khaldoun: Reduction of Water Loss in Karak Authority Distribution System Using GIS Technology (Unpublished master's thesis). Mutah University, Karak. Retrieved from http://search.mandumah.com/Record/783269. 2015.

[5] Alkelani M. T. M. Z, and Awad. M. Prediction of Water Demand in North of Palestine Using Artificial Neural Networks. http://search.mandumah.com/record/1019967. 2018.

[6] Al-Nairab, Abdel-Rahman Farid Abdel-Rahman, & Tayeh, Bassam Abdel-Rahman Othman. Challenges Facing Municipalities to Provide Infrastructure Services in the Gaza Strip. Retrieved from http://search.mandumah.com/Record/1010099. 2018.

[7] Allan Lambert, Richard Taylor: water New Zealand. Giz: 2011 Guide lines for water loss. Reduction Frankfort, Germany. 2010.

[8] Awadallah W, Owaiwi M. Hydrogeology, Hydrology & Hydrochemistry of Dug Wells & Springs in the Western Hebron District Basin. Palestinian Hydrology Group, Hebron, Palestine. 2004.

[9] Bill Kingdom, Roland Liemberger, Philippe Marin: The challenge of reducing non-revenue water (NRW) In developing Countries, How the private sector Can help: ALook at Performance-Based Service Contracting, The world Bank group. 2012.

[10] Dura Municipality. Statistics on drinking water, wastewater, and solid waste management in Dura city and Al-Fawwar camp (2014–2024 data) [Unpublished raw data]. Dura Municipality, Hebron, Palestine.2024.

[11] Fourth Geneva Convention. Geneva Convention Relative to the Protection of Civilian Persons in Time of War. 1949.

[12] J. Sirajudeen Arul Manikandan and V. Manivel. Water Quality Index of Ground Water around Ampikapuram area near Uyyakondan channel Tiruchirappalli Tamil Nadu, Archives of Applied Science Research. 2013;5(3)21–26.

[13] Oslo II Agreement. Israeli-Palestinian Interim Agreement on the West Bank and the Gaza Strip.1995.

[14] Palestinian News and Information Agency (WAFA, 2000): Website. Ramallah, Palestine.

[15] Palestinian Water Authority. Strategic Plan 2020–2022+23. Ramallah, Palestine.2020.

[16] Rammal FY, El Khazan B. Social vulnerability related to sanitation and its connection to environmental injustice in the West Bank and Gaza Strip, Palestine. J Hum Soc Sci. 2022;6(2):98–131.

[17] Saleh MKS, Rattroot A. Prediction of Pipes Break in Water Distribution System Using Data Mining Tools: Case Study Nablus Municipality (Unpublished master's thesis). Arab American University, Jenin. 2018. Retrieved from http://search.mandumah.com/record/1020572

[18] Selby J. Cooperation, domination and colonization: the Israeli-Palestinian Joint Water Committee. Water Altern. 2013;6(1):1–24.

[19] Trottier J. Hydropolitics in the West Bank and Gaza Strip. Jerusalem: PASSIA; 1999.

[20] United Nations. Transforming our world: The 2030 agenda for sustainable development. United Nations. 2015.

[21] United Nations General Assembly. (2010): Resolution 64/292: The human right to water and sanitation. A/RES/64/292.

[22] Water Sector Regulatory Council (WSRC). Performance Report of Water and Wastewater Service Providers in Palestine 2018. Ramallah, Palestine: Water Sector Regulatory Council. 2018.

[23] Weinthal E, Sowers J. Targeting infrastructure and livelihoods in the West Bank and Gaza. Int Aff. 2019;95(2):319–40.

[24] World Health Organization, WHO. Guidelines for drinking-water quality: fourth edition incorporating the first addendum. Geneva: World Health Organization; 2017. 28759192

[25] Zeitoun M. Power and water in the Middle East: The hidden politics of the Palestinian-Israeli water conflict. London: I.B. Tauris; 2008.

